# Diagnosis of drowning using postmortem computed tomography combined with endoscopic autopsy: A case report

**DOI:** 10.1097/MD.0000000000019182

**Published:** 2020-03-13

**Authors:** Zhuoqun Wang, Kaijun Ma, Donghua Zou, Ningguo Liu, Zhengdong Li, Yu Shao, Yijiu Chen

**Affiliations:** a Shanghai Key Laboratory of Forensic Medicine, Shanghai Forensic Service Platform, Academy of Forensic Science, Shanghai; b Shanghai Key Laboratory of Crime Scene Evidence, China.

**Keywords:** postmortem computed tomography, targeted coronary postmortem computed tomography angiography, endoscopic autopsy, postmortem laparoscopic examination, drowning

## Abstract

**Rationale:**

Postmortem forensic imaging technologies provide a noninvasive/minimally invasive approach for imaging of internal organ structures of the human body to detect injuries, diseases, and other morphologic changes. Currently, postmortem forensic imaging methods have been widely used in determination of the cause of death. However, these methods do not allow histologic examinations. Endoscopic autopsy emerged in the 1990s. Thoracoscopy and laparoscopy are mainly used to examine organs and tissues in the thoracic and abdominal cavity. Target tissues are also sampled for histologic examination. By combining postmortem forensic imaging with endoscopic autopsy, comprehensive examination of the corpse, organs, and sampling for histologic examination can be carried out.

**Patient concerns:**

A 34-year-old woman was witnessed jumping into the river, sinking after struggling in the water. The body was found 24 hours later and confirmed with no vital signs. No preexisting medical conditions were known.

**Diagnosis, interventions, and outcomes:**

Postmortem computed tomography, target coronary postmortem computed tomography angiography, and endoscopic autopsy were performed before conventional autopsy. Laparoscopic examination was used to examine the abdominal organs. The diaphragm and pericardium were cut open from the abdominal cavity to allow access to the examination of lungs and heart. Tissue samples were collected from various organs for histologic examination, and a diatom test was carried out on lung samples. Postmortem computed tomography revealed fluid in the paranasal sinuses, airways, stomach, and duodenum; emphysema aquosum; and mosaic pattern of the lung parenchyma. Endoscopic examination additionally detected Paltauf spots. The results were consistent with those of conventional autopsy. Histologic examination revealed pulmonary congestion, pulmonary edema, pulmonary emphysema, pulmonary hemorrhage, and congestion in multiple organs such as the liver, spleen, and kidneys. Diatoms were detected in lung tissues, which were identical in morphology to diatoms in water samples collected from the scene. The cause of death was determined as drowning.

**Conclusion:**

Combining forensic imaging and endoscopic autopsy for postmortem examination yields a more comprehensive and scientific finding, and the combination is minimally invasive and more acceptable to the family members. This method can be used as an alternative for conventional autopsy under specific circumstances.

## 1 Introduction

Postmortem forensic imaging technologies provide a noninvasive/minimally invasive approach for imaging of internal organ structures of the human body to detect injuries, diseases, and other morphologic changes. Currently, postmortem forensic imaging methods, such as postmortem computed tomography (PMCT), postmortem magnetic resonance imaging (PMMR), and postmortem computed tomography angiography (PMCTA) have been widely used in crime scene reconstruction, injury detection, and determination of the cause of death.^[1]^ However, these techniques still have some shortcomings. PMCT and PMMR have limited sensitivity for detecting certain injuries and lesions in different human tissues, and there is a possibility of missed diagnosis. In addition, these methods do not allow histologic examinations.^[1]^ Endoscopic autopsy emerged in the 1990s; in this technique, thoracoscopy and laparoscopy are mainly used to examine organs and tissues in the thoracic and abdominal cavity. Target tissues are also sampled for subsequent histologic examination. Endoscopic autopsy has been proven to have high sensitivity and detection rates for injuries and lesions in postmortem examination. Compared with conventional autopsy, endoscopic autopsy has advantages such as simple operation and less time consuming. This minimally invasive technique is more acceptable to the family members of the deceased and can be used as an alternative for conventional autopsy under specific circumstances.^[2–7]^ However, endoscopic autopsy also has some shortcomings. The nature of endoscopic autopsy is identical to that of conventional autopsy, and the process is still examiner dependent. Moreover, there is a possibility of a lack of purpose, a lack of focus, and incomplete examination. In addition, endoscopic autopsy also has a lower detection rate for certain lesions and poor capability for examinations of certain anatomical structures and organs.^[2–7]^ By combining postmortem forensic imaging with endoscopic autopsy, comprehensive examination of the corpse, in-depth examination of target organs, and sampling for histologic examination can be carried out. The present study reported a case of drowning diagnosed via PMCT, targeted coronary PMCTA, and endoscopic autopsy and confirmed via conventional autopsy.

## 2 Case report

A 34-year-old woman was witnessed jumping into the river from the bridge, sinking after struggling in the water. The body was found 24 hours later and confirmed with no vital signs. No preexisting medical conditions were known. Postmortem examination was carried out after 5 days. The examining procedure included external examination, PMCT, targeted coronary PMCTA, endoscopic autopsy, conventional autopsy, histologic examination, toxicology analysis, and diatom test. This study was approved by the Academic Committee of the Academy of Forensic Science. Written informed consent to publish the case details were obtained from the victim's family.

### 2.1 PMCT and targeted coronary PMCTA

Once the standard external examination was completed, PMCT was conducted. The entire body was scanned using a 40-slice multislice CT system (Definition AS; Siemens Healthineers, Erlangen, Germany). Image review and 3-dimensional reconstructions were performed on a CT workstation (Syngo Imaging XS; Siemens Healthineers). Settings were described in detail previously.^[8,9]^

The angiography protocol was also as described previously.^[10]^ An incision was made into the left common carotid artery, and a 3-way urinary catheter with a 30-mL balloon was inserted. The position of the catheter tip was determined using CT to reach a position exactly above the aortic valve. The balloon was then fully inflated. After collecting blood samples for toxicologic analyses, 150 mL of contrast medium (diatrizoate meglumine and normal saline [0.9%] at 1:10 ratio) was injected manually at a rate of 50 mL/8 s. Scanning was performed directly after administering the contrast medium.

### 2.2 Endoscopic autopsy

An incision was made at the navel to create a working space within the abdominal cavity using carbon dioxide through a 10-mm trocar before inserting a telescope (10 mm, 30°) (Richard-wolf, Berlin, Germany). Incisions were made at the bilateral midclavicular lines and 1 cm below the costal margin. Two 10 mm trocars were used to insert the operating equipment. The abdominal organs, including the liver, gallbladder, spleen, kidneys, pancreas, stomach, and intestines, were examined. Samples for histologic examination were collected from these organs. The diaphragm was cut from the abdominal cavity to access the thoracic cavities for examinations of both lungs, and samples were collected. Lung samples for diatom tests were also collected. The pericardium was cut open, and the heart was examined, and samples were collected.

### 2.3 Autopsy and other analyses

Conventional autopsy was performed approximately 30 minutes after the endoscopic autopsy. During the autopsy, external and internal examinations of the body were performed. Histologic samples of the organs were subjected to hematoxylin and eosin staining. Blood was sent for toxicologic analyses. Diatom tests of the lung samples and on-site water samples were performed.

## 3 Results

**Figure 1:**
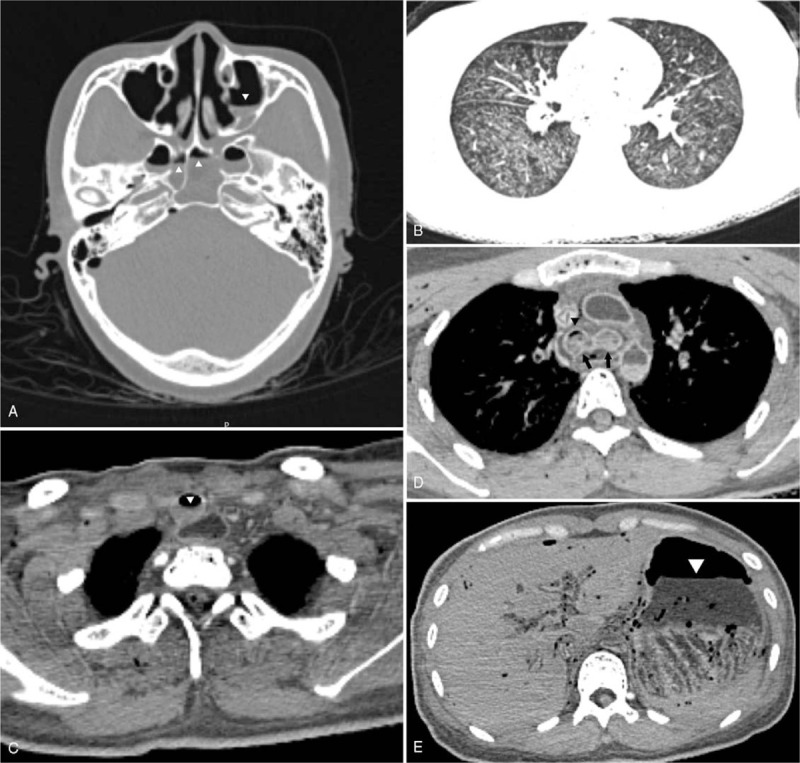
Postmortem computed tomography shows (A) air/fluid in the sphenoidal sinus (arrowheads) and the left maxillary sinus (arrowhead); (B) mosaic patterns of the lungs; (C, D) air/fluid in the trachea (arrowhead) and fluid in the main bronchi (arrows) with an air/fluid level on the right side (arrowhead); and (E) air/fluid in a distended stomach (arrowhead).

**Figure 2:**
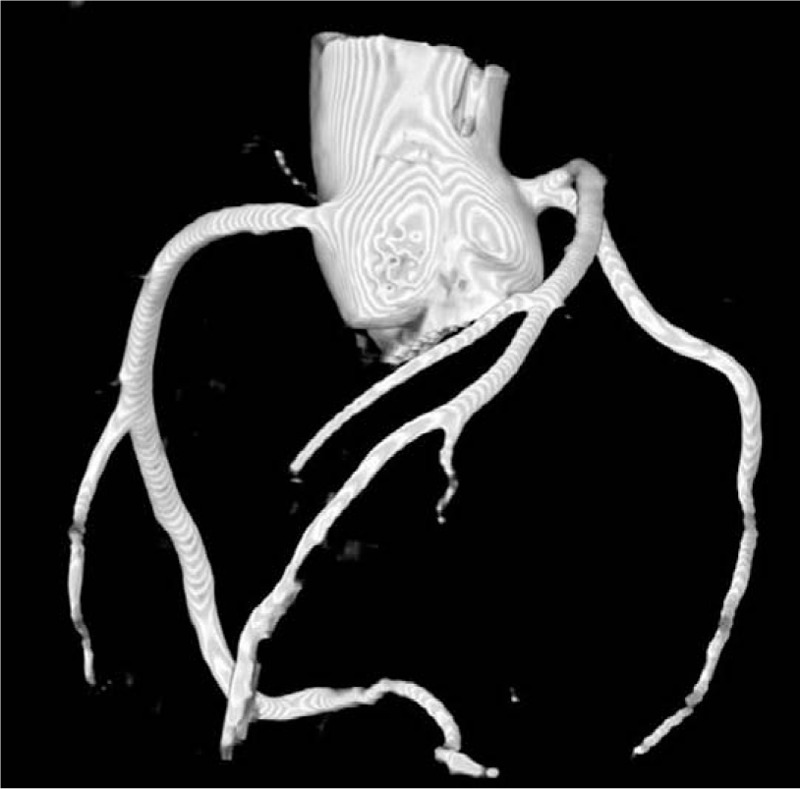
Three-dimensional reconstruction of coronary postmortem computed tomography angiography shows no abnormalities in the various major branches.

The PMCT and PMCTA, endoscopic autopsy, and conventional autopsy took 1, 2, and 2 hours, respectively. PMCT results (Fig. 1) showed fluid in the bilateral maxillary sinuses and sphenoidal sinuses, trachea, and bronchi; small amounts of bilateral pleural effusion; diffuse mosaic patterns of hyperdense shadows in both lungs; pulmonary emphysema; gastric and duodenal dilatation and effusion; and small amounts of pericardial effusion. Coronary PMCTA results (Fig. 2) showed that the left and right coronary arteries and various branches were intact, and no stenosis was observed.

**Figure 3:**
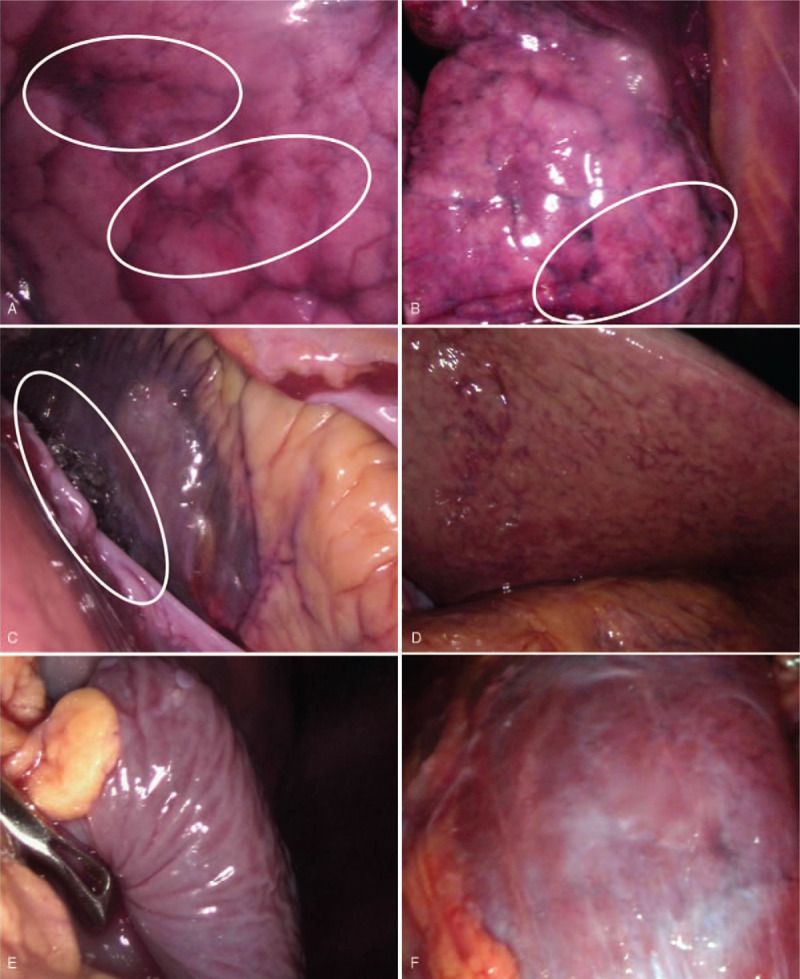
Endoscopic autopsy views of the (A, B) lungs, (C) heart, (D) liver, (E) spleen, and (F) kidney. Note the hemorrhage spots in the lungs, that is, Paltauf spots (circles) and pericardial effusion (circles).

Endoscopic autopsy results (Fig. 3) showed pulmonary emphysema, Paltauf spots (i.e., cloudy-shaped bleeding spots at the lower lobes of both lungs), and small amounts of pericardial effusion. No apparent abnormalities in other organs were observed.

**Figure 4:**
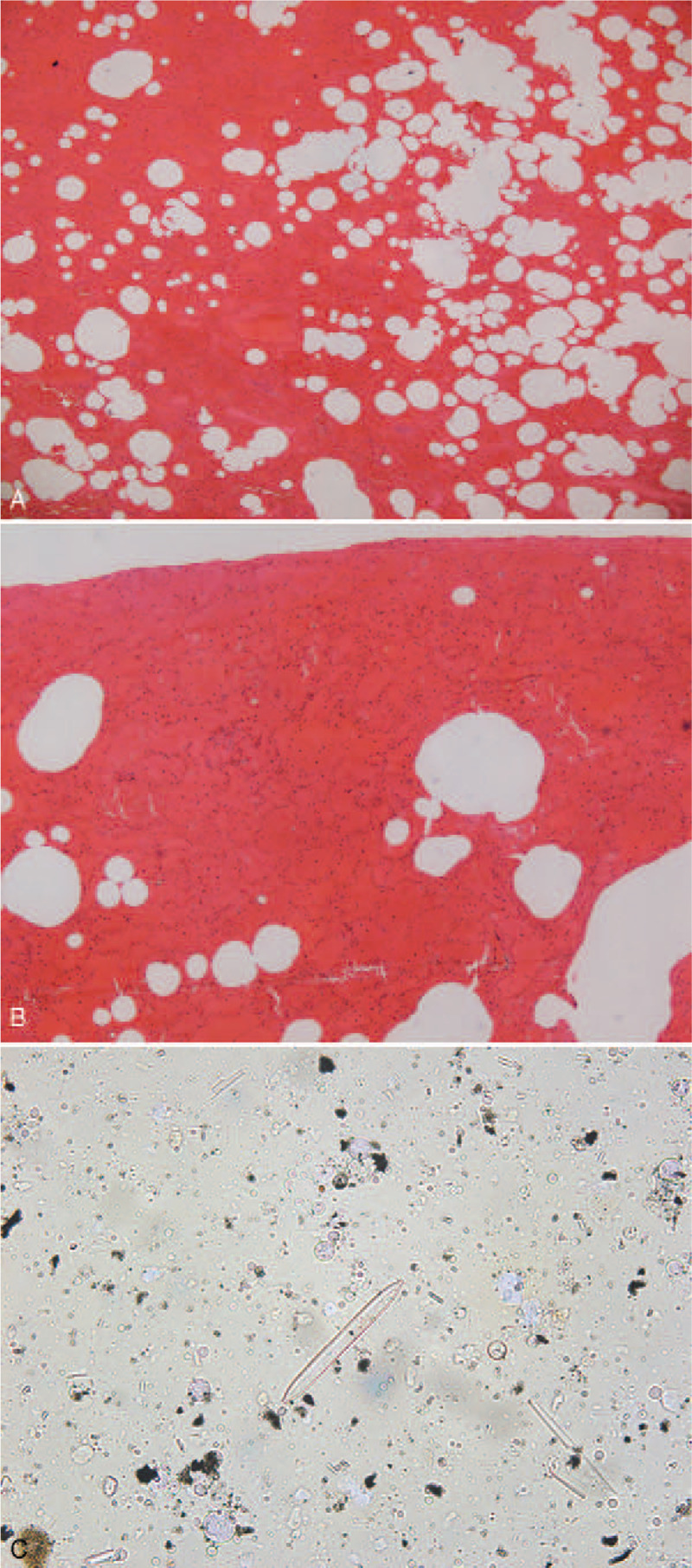
Histologic examinations and diatom tests show (A) pulmonary edema accompanied with pulmonary emphysema; (B) pulmonary hemorrhage; and (C) diatoms in lung tissues.

During external examination, the body was of medium body shape and without developmental malformations. Cyanosis of the lips and bilateral fingernails were observed. The skin of both feet was pale and wrinkled. A 1 cm × 0.5 cm skin abrasion was present at the back of the right hand. No apparent injuries were observed in the rest of the body. Internal examination revealed small amounts of bilateral pleural effusion, with the left and right lungs weighing 424 and 549 g, respectively. Pulmonary emphysema was present. Paltauf spots in the lower lobes of both lungs were observed. Small amounts of pericardial effusion were observed; the weight of the heart was 235 g; and no apparent abnormalities were observed. The stomach contained 400 mL of a pale-brown liquid. No apparent abnormalities were observed in other organs. Histologic examination (Fig. 4) revealed pulmonary congestion, pulmonary edema, pulmonary emphysema, pulmonary hemorrhage, and congestion in multiple organs such as the liver, spleen, and kidneys.

Toxicologic results were negative for alcohol and common drugs. Various diatoms were detected in the lung tissue, consistent with the morphology of the diatoms in the water samples.

The cause of death was concluded as drowning.

## 4 Discussion

Forensic autopsy is recognized as the gold standard for establishing a clear cause of death. However, conventional autopsy is a destructive examination, and there have been resistance and objection from the family of the deceased due to the unacceptability of the cosmetic effects of large incisions and concerns regarding organ retention and sometimes also due to some religious and cultural traditions.^[1,11,12]^

Postmortem forensic imaging uses computerized and medical imaging techniques to examine injuries and lesions inside the human body. Compared with conventional autopsy, postmortem forensic imaging has advantages such as noninvasiveness/minimally invasiveness, reproducibility, intuitiveness, and objectiveness. Currently, PMCT, PMMR, and PMCTA are regarded as helpful for conventional autopsy or as alternative methods in circumstances in which autopsy is difficult to carry out.^[1,11,13]^ However, postmortem forensic imaging also has certain limitations. The quality of image acquired strongly depends on device performance, scanning parameters, body condition, the performance of the device-dependent software, and operators’ personal judgments through the digital postprocessing.^[8]^ The image cannot present the true color of tissues, and artifacts may appear during the scanning. In addition, it is difficult to perform a histologic examination of the lesions detected via imaging techniques.^[1]^

Thoracoscopy and laparoscopy are commonly used techniques in minimally invasive surgeries in clinical practice and are widely used in general surgery, urology, gynecology, and obstetrics surgeries. These techniques have advantages such as small trauma, low infection rate, and faster recovery.^[14]^ Since the 1990s, thoracoscopy and laparoscopy have been used in forensic postmortem examination and determination of the cause of death.^[2–7]^ Relevant studies have shown that endoscopic autopsy has a high detection rate for hemothorax; hemoperitoneum; aortic injury; and injuries in the liver, spleen, and diaphragm. Endoscopic autopsy analysis of the cause of death according to endoscopic autopsy findings is accurate, and sampling from various organs in the thoracic and abdominal cavities under endoscopy is successful. This method can be used as an alternative when it is difficult to carry out conventional autopsy or in regions where there are no organizations or conditions to conduct conventional autopsy.^[4,5]^ However, endoscopic autopsy has some shortcomings, such as lower detection rate for retroperitoneal, retromediastinal, and internal injuries and lesions of the gastrointestinal system. Moreover, similar with conventional autopsy, there remains a possibility of a lack of objectives, a lack of focus, and incomplete examination when the endoscope is used alone in endoscopic autopsy.^[3,4,6,7]^

In the present case, postmortem forensic imaging and endoscopic autopsy were combined. PMCT was used for comprehensive examination of the deceased, examine suspected lesions and injury sites, and perform accurate localization. This provided focus for subsequent endoscopic autopsy for targeted examinations. The succeeding endoscopy was employed to examine and sample organs in the thoracic and abdominal cavities for histologic examination and diatom tests. The endoscopic and histologic examinations could complement the shortcomings of imaging techniques, and the examination results could also confirm the findings observed in PMCT. The combination of the 2 methods fully utilizes the strengths of both techniques and retains the advantage of minimal invasion, which is more acceptable to family members.

In the present case, PMCT results included fluid in the paranasal sinuses, airways, stomach, and duodenum; emphysema aquosum; and mosaic patterns of the lung parenchyma. These are typical PMCT findings of drowning.^[15,16]^ Endoscopy can be used to examine the actual color and texture of organs, which cannot be reflected in CT images. In this case, endoscopy examinations found Paltauf spots in the lower lobes of both lungs, and histopathology examinations confirmed pulmonary hemorrhage. These hemorrhage spots, which are considered to be among the typical signs of drowning, were not observed in CT images. Endoscopic examinations confirmed the PMCT findings of small amounts of bilateral pleural effusion and pericardial effusion. Lung tissues collected via endoscopy were used for diatom tests, and the diatoms detected were identical to those in water samples, which aided in the diagnosis of drowning.

Although the drowning of the deceased was witnessed, and PMCT results support drowning as a cause of death, there is still a need to exclude the possibility of sudden death due to cardiac and intracranial diseases. In the present case, PMCT did not detect intracranial hemorrhage, tumors, cerebral infarction, and other changes. Moreover, no heart rupture, cardiac hypertrophy, dilated ventricles, and myocardial infarction were observed. However, it is difficult to detect coronary artery lesions via PMCT. Therefore, PMCTA must be performed. In addition, we employed endoscopic autopsy to obtain samples, conduct endoscopic examinations, and exclude the possibility of potentially fatal diseases such as myocarditis and cardiomyopathy, which cannot be diagnosed via PMCT alone.

Studies showed that combining postmortem forensic imaging and conventional autopsy will yield a more objective finding compared with that obtained via conventional autopsy alone because the findings from these 2 methods can be compared and analyzed.^[1,13]^ Currently, there are numerous cases in which postmortem forensic imaging and autopsy findings were combined to determine the cause of death. In the current case, endoscopic autopsy served the purpose of conventional autopsy. Endoscopic autopsy can be used to examine organs in the thoracic and abdominal cavity, particularly in both lungs. In addition, samples can be collected for histologic examination and diatom tests. Postmortem forensic imaging, endoscopic autopsy, and histologic examination results were consistent with those of conventional autopsy, and the cause of death according to findings obtained from these modalities was accurately determined. The results from the combined use of postmortem forensic imaging and endoscopic autopsy are more comprehensive and consumed for the examination was slightly higher than that of conventional autopsy alone, the minimal invasiveness of these techniques make it an alternative method for cases in which conducting an autopsy can be difficult. However, there remain some drawbacks to this method; for example, the endoscope currently utilized cannot be used for intracranial examination, but this can be solved by performing intracranial endoscopy.

## 5 Conclusion

In the present study, PMCT and endoscopic autopsy were used to achieve a successful diagnosis of drowning, while retaining minimal invasiveness. The combination of postmortem forensic imaging and endoscopic autopsy yields more comprehensive and scientific findings than any single method and can be used as an alternative for conventional autopsy under certain conditions.

## Author contributions

**Conceptualization:** Donghua Zou, Yu Shao, Yijiu Chen.

**Investigation:** Kaijun Ma.

**Methodology:** Zhengdong Li.

**Supervision:** Ningguo Liu, Yijiu Chen.

**Visualization:** Ningguo Liu.

**Writing – original draft:** Zhuoqun Wang.

**Writing – review & editing:** Zhengdong Li, Yu Shao.
